# Genome-wide association analysis identified molecular markers and candidate genes for flower traits in Chinese orchid (*Cymbidium sinense*)

**DOI:** 10.1093/hr/uhad206

**Published:** 2023-10-13

**Authors:** Fengxi Yang, Yudi Guo, Jie Li, Chuqiao Lu, Yonglu Wei, Jie Gao, Qi Xie, Jianpeng Jin, Genfa Zhu

**Affiliations:** Guangdong Key Laboratory of Ornamental Plant Germplasm Innovation and Utilization, Environmental Horticulture Research Institute, Guangdong Academy of Agricultural Sciences, Guangzhou 510640, China; Guangdong Key Laboratory of Ornamental Plant Germplasm Innovation and Utilization, Environmental Horticulture Research Institute, Guangdong Academy of Agricultural Sciences, Guangzhou 510640, China; Guangdong Key Laboratory of Ornamental Plant Germplasm Innovation and Utilization, Environmental Horticulture Research Institute, Guangdong Academy of Agricultural Sciences, Guangzhou 510640, China; Guangdong Key Laboratory of Ornamental Plant Germplasm Innovation and Utilization, Environmental Horticulture Research Institute, Guangdong Academy of Agricultural Sciences, Guangzhou 510640, China; Guangdong Key Laboratory of Ornamental Plant Germplasm Innovation and Utilization, Environmental Horticulture Research Institute, Guangdong Academy of Agricultural Sciences, Guangzhou 510640, China; Guangdong Key Laboratory of Ornamental Plant Germplasm Innovation and Utilization, Environmental Horticulture Research Institute, Guangdong Academy of Agricultural Sciences, Guangzhou 510640, China; Guangdong Key Laboratory of Ornamental Plant Germplasm Innovation and Utilization, Environmental Horticulture Research Institute, Guangdong Academy of Agricultural Sciences, Guangzhou 510640, China; Guangdong Key Laboratory of Ornamental Plant Germplasm Innovation and Utilization, Environmental Horticulture Research Institute, Guangdong Academy of Agricultural Sciences, Guangzhou 510640, China; Guangdong Key Laboratory of Ornamental Plant Germplasm Innovation and Utilization, Environmental Horticulture Research Institute, Guangdong Academy of Agricultural Sciences, Guangzhou 510640, China

## Abstract

The orchid, the champagne of flowers, brings luxury, elegance, and novelty to nature. *Cymbidium sinense* is a symbol of gigantic floral variability on account of wavering shapes and sizes of floral organs, although marker–trait association (MTA) has not been studied for its floral traits. We evaluated markers associated with 14 floral traits of *C. sinense* through a genome-wide association study (GWAS) of 195 accessions. A total of 65 318 522 single-nucleotide polymorphisms (SNPs) and 3 906 176 insertion/deletion (InDel) events were identified through genotyping-by-sequencing. Among these, 4694 potential SNPs and 477 InDels were identified as MTAs at −log_10_ *P* > 5. The genes related to these SNPs and InDels were largely associated with floral regulators, hormonal pathways, cell division, and metabolism, playing essential roles in tailoring floral morphology. Moreover, 20 candidate SNPs/InDels linked to 11 genes were verified, 8 of which were situated on exons, one was located in the 5′-UTR and two were positioned in introns. Here, the multitepal trait-related gene *RABBIT EARS* (*RBE*) was found to be the most crucial gene. We analyzed the role of *CsRBE* in the regulation of flower-related genes via efficient transient overexpression in *C. sinense* protoplasts, and found that the floral homeotic genes *CsAP3* and *CsPI*, as well as organ boundary regulators, including *CsCUC* and *CsTCP* genes, were regulated by *CsRBE*. Thus, we obtained key gene loci for important ornamental traits of orchids using genome-wide association analysis of populations with natural variation. The findings of this study can do a great deal to expedite orchid breeding programs for shape variability.

## Introduction

The Orchidaceae falls among the largest angiosperm families. Based on the Plants of the World Online (POWO) resource, there are 31 069 accepted orchid species. *Cymbidium*, with 104 species listed by POWO and more than 15 000 popular commercial hybrids recorded in the horticultural database of the Royal Horticultural Society (RHS), stands out as the orchid genus of the highest renown. *Cymbidium*s are among the earliest orchid species to be cultivated [1]. Recently, the whole-genome sequencing of *Cymbidium* species has broadened the breeding scope for functional genomics [2, 3]. However, marker-assisted gene mining has not been used for *Cymbidium* breeding.

Molecular markers are an indispensable tool during marker-assisted selection (MAS) to support preferred phenotypic traits in crop selection. Single-nucleotide polymorphism (SNP) markers can be effectively identified through ultra-throughput next-generation sequencing, which has revolutionized selective-trait breeding [4, 5]. For large-genome crops, next-generation sequencing has been broadened by establishing genotyping-by-sequencing (GBS), which is used for the sequencing of pooled samples to identify molecular markers [6]. GBS, being an ultimate MAS and cost-effective, has proven to be effective for GWAS [7]. For large-scale populations, GBS is an excellent choice for selective-trait breeding and it has been successfully applied to many commercial crops, such as *Lactuca sativa* [8], *Glycine max* [9], *Brassica napus* [10], and *Zea mays* [11]. It has recently been used for an orchid *Phalaenopsis equestris* [12].

For *Cymbidium* breeding, *C. sinense* is an ideal source and a popular breeding material in nurseries. It is recognized by its dark and attractive foliage, and elegant and scented flowers. With more than 1000 natural variants, *C. sinense* is an ideal specimen to trace the evolutionary history of phenotypic traits of orchids [2]. It has small flowers of ~2 inches in diameter. Among the current varieties/hybrids, significant diversity is found for flower size, flower number, sepal shape, petal shape, and lip shape. Therefore, it is of interest to investigate the visual floral traits using GWAS and MAS. The thoroughly annotated and high-quality reference genome of *C. sinense* is a great source for SNP calling. However, GWAS implementation remains to be established in *Cymbidium* orchids.

Therefore, we used 195 *C. sinense* resources to study 14 phenotypes related to flower size and the number and morphology of tepals. We used SNPs to build a genetic map of *C. sinense* and discovered the relationships by linking the SNPs with the floral traits to recognize quantitative trait loci (QTLs) contributing to 14 diverse floral traits in *Cymbidium*. This pioneer report thus explores novel loci associated with flower size and tepal morphology-related traits of *Cymbidium* orchids.

## Results

### Phenotypic variation of flower traits in the *Cymbidium sinense* collections

**Figure 1:**
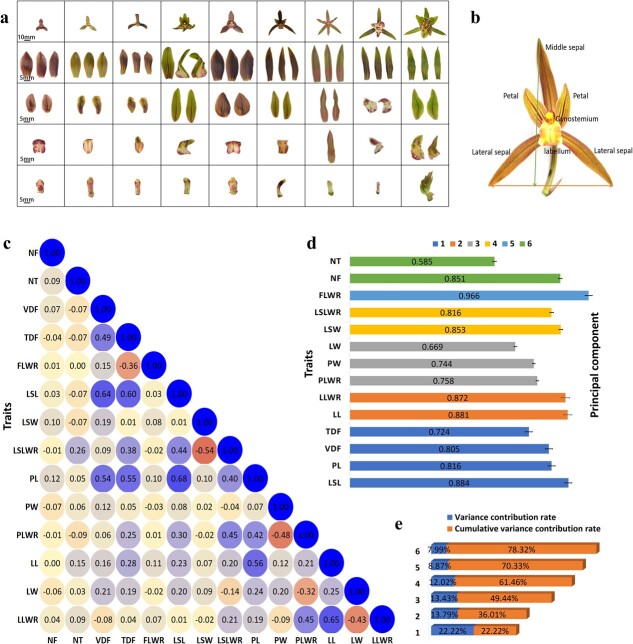
Floral organ morphology correlation analysis among 14 flower traits [number of flowers (NF), number of tepals (NT), vertical diameter of flower (VDF), transverse diameter of flower (TDF), flower length to width ratio (FLWR), lateral sepal length (LSL), lateral sepal width (LSW), lateral sepal length to width ratio (LSLWR), petal length (PL), petal width (PW), petal length to width ratio (PLWR), lip length (LL), lip width (LW), and lip length to width ratio (LLWR)]. **a** Flower shapes and flower organ diversification of *C. sinense.* First row: morphological structure of the entire flower; scale bar = 10 mm. Second row: three sepals in the first whorl, including one middle sepal and two lateral sepals. Third row: two petals in the second whorl. Fourth row: unique shape of the labellum. Fifth row: shape of the gynostemium. Illustrations are arranged from left to right according to flower organ size, from small to large. **b** Typical *C. sinense* flower with four floral whorls. The vertical arrow shows the vertical diameter of the flower and the horizontal arrow shows the transverse diameter of the flower. **c** Correlation analysis among 14 floral traits. **d** Principal component selection based on eigenvalue. **e** Variance and cumulative variance contribution rates.

A normal flower of *C. sinense* has three sepals and three petals in the first and second whorls, respectively. A striking similarity is found between two of the petals. However, the third petal, called the labellum (lip), is significantly evolved, showing a curled and inverted triangular shape. The highly fused male and female reproductive organs make a gynostemium (column) at the center. It is worth noting that *C. sinense* showcases an extensive array of naturally occurring variations in flower organ types (Fig. 1a and b). The size and shape of floral organs vary among different accessions. As shown in Table 1, the length, width, and length/width ratio of the perianth show significant differences, and can be classified into lotus-like perianth, gynostemium-like perianth, labellum-like petal, multi-perianth flower, and null-labellum flower, according to floral organ transformation and/or reversion. For example, when the lip develops degenerately and does not curl, it forms lotus-like petals, whereas lip-like petals are formed when the lip strengthens and expands. In severe cases, the lip is missing in a null-labellum flower (Supplementary Data Fig. S1).

From 195 plant resources of a natural variation population, a total of 14 phenotypes were assessed, which were related to flower size and the number and morphology of tepals. These traits included number of flowers (NF), number of tepals (NT), vertical diameter of the flower (VDF), transverse diameter of the flower (TDF), flower length to width ratio (FLWR), lateral sepal length (LSL), lateral sepal width (LSW), lateral sepal length to width ratio (LSLWR), petal length (PL), petal width (PW), petal length to width ratio (PLWR), lip length (LL), lip width (LW), and lip length to width ratio (LLWR).

The parameters of individual flower organs exhibited significant variation among the 195 accessions, some showing differences exceeding 20-fold (Table 1 and Supplementary Data Table S1). The raceme produced an average of 7.90 flowers, ranging from 2 to 16. Among the 195 accessions, 42 varieties produced more than 10 flowers, while 23 varieties had fewer than 5 flowers, flower number showing a relatively large standard deviation of 2.74. The NT ranged from 3 to 20 with a mean of 6.28 (Table 1). Most of the flowers contained six tepals and 10 varieties had more than eight tepals. The parameters of individual flower organs also varied distinctly among the 195 accessions, with a difference of >10-fold. Particularly, the difference between the minimum and maximum values of LSW, LSLWR, and PW was >20-fold. The coefficient of variation (CV) ranged from 0.18 to 0.86. The CV of petal length was the smallest and the CV of lateral sepal width was the largest. The diversity index was between 4.92 and 5.08, reflecting the significant polymorphism among the determinants of flower type (Table 1 and Supplementary Data Table S1).

### Relationships and principal components among all characteristics

Correlation analysis of 14 traits inferred significant correlations among 32 pairs (correlation coefficient >0.2 and <−0.02) (Fig. 1c). Of these, 27 pairs showed positive correlations and only 5 pairs demonstrated significantly negative correlations. LSL exhibited highly positive correlations with PL (0.68), VDF (0.64), and TDF (0.6), followed by LL with highly positive correlations with LLWR (0.65) and PL (0.56). The most negatively correlated pairs included LSW and LSLWR (−0.56), PW and PLWR (−0.48), and LW and LLWR (−0.43) (Fig. 1c).

Following the principle of eigenvalue >1, six principal components were selected (Fig. 1d). These components included the highly variable, either positively or negatively correlated, traits. Component 1 contained more variables (LSL, PL, VDF, and TDF) than other components, while component 5 contained only one variable (FLWR). The lowest variance contribution rate was shown by component 6; however, its cumulative contribution rate was the greatest (78.32%), compared with other components (Fig. 1e). Six main feature vectors were selected, including LSL, LL, PLWR, LSW, FLWR, and NF, which could be used as important traits in breeding evaluation of new varieties.

### Genome-wide SNP identification and genetic relationships

**Figure 2:**
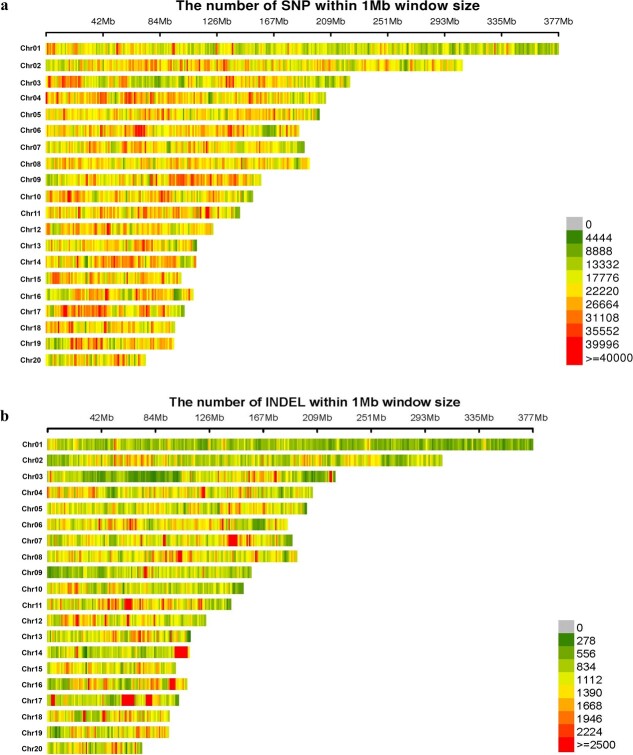
Distribution of SNPs (**a**) and InDels (**b**) on each chromosome of *C. sinense*. The X-coordinate is the length of each chromosome, each stripe represents a chromosome, and the dark red color indicates a higher density of SNPs per 1 Mb in the genome of *C. sinense*.

We used an HaeIII+Hpy166II enzyme digestion assay to define Specific-Locus Amplified Fragment (SLAF) tags for sequences with the length of 500–550 cleaved fragments. Using 195 accessions, 675 761 SLAF tags were produced, with an average sequencing depth of 10.01× per sample (Supplementary Data Table S2). Among these, 14 457 SLAF tags were polymorphic. Using the reference of the highest copy number per SLAF, 65 318 522 population SNPs and 3 906 176 InDels were identified in 195 accessions. The SNPs were dispersed over 20 chromosomes, covering a region of 3246.66 Mb (Fig. 2a). The SNP distribution ranged from 72.95 Mb on chromosome 20 to 376.78 Mb on chromosome 1 (Table 2). For individual chromosomes, the number of SNPs fluctuated from 1 540 306 (chromosome 20) to 6 632 474 (chromosome 1). Additionally, an average marker interval of 0.00045 Mb was observed across all the chromosomes, ranging from 0.00004 Mb (chromosome 17) to 0.000057 Mb (chromosome 1). The largest gap, of 0.069 Mb, was found on chromosomes 1 and 8 (Table 2).

In addition to SNPs, we also detected a total of 3 906 176 InDel markers (Table 3), covering a region of 3246.68 Mb on 20 chromosomes (Fig. 2b). For individual chromosomes, the InDel distribution ranged from 85 082 (chromosome 20) to 324 381 (chromosome 2). The average marker interval ranged from 0.00071 Mb (chromosome 11) to 0.00130 Mb (chromosome 1) (Table 3).

**Figure 3:**
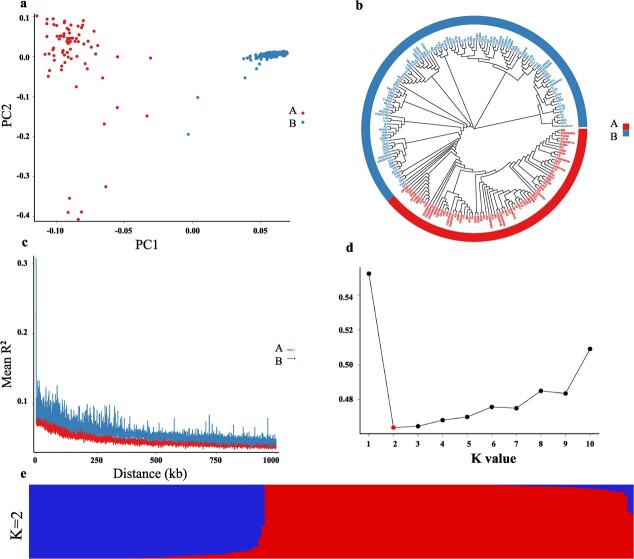
**a** PCA plot of the first two components (PC1 and PC2). **b** GWAS populations based on a whole-genome filtered high-quality SNP dataset. **c** Decay of LD in two clusters. **d** Plot of Δ*K* value with *K* from 1 to 10 based on the Evanno test. **e** Population structure analysis with a different number of clusters (*K* = 2).

PCA (principal component analysis) showed that two components (PC1 and PC2) could clearly divide 195 *C. sinense* accessions into two distinct groups (Fig. 3a). We used the neighbor-joining method for the clustering of 195 accessions. All the accessions were clustered into two distinguishable groups (Fig. 3b), justifying the PCA predictions. Linkage disequilibrium (LD) determines the resolution of trait mapping. An *R*^2^ value <0.1 shows higher mapping resolution for diverse germplasm resources [13]. Analysis of LD decay of all the collections showed that both the clusters had a significant *R*^2^ value <0.1, indicating a low level of LD in the genome (Fig. 3c). The genetic structures of the 195 accessions were analyzed with different clusters (*K* values from 1–10) using the rate of cross-validation error (Fig. 3d). The lowest cross-validation error rate was observed at *K* = 2 (Fig. 3e), showing that the 195 accessions can indeed be grouped into two clusters.

### Identification of marker–trait associations for flower traits

**Figure 4:**
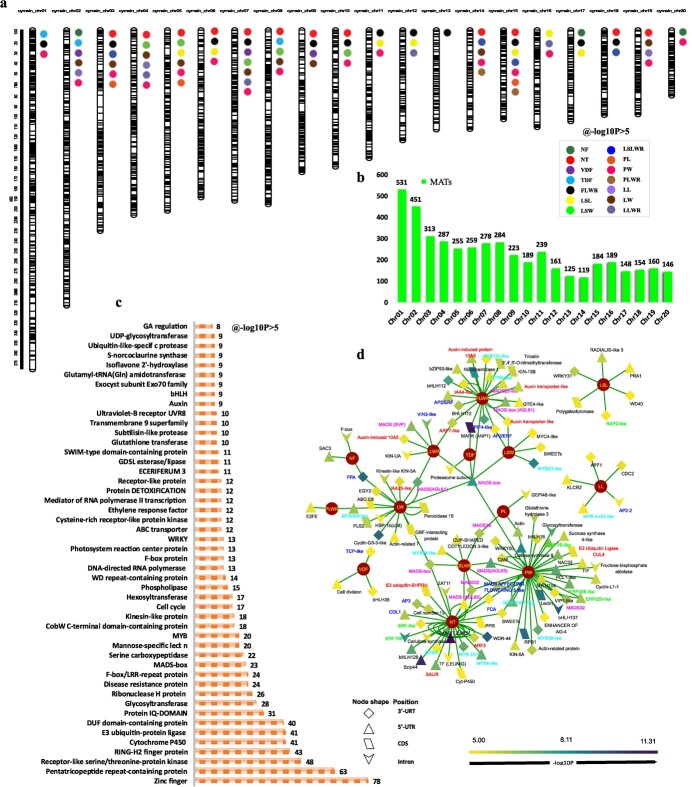
**a** Physical map positions of the MTAs detected in this study for flower traits. The dark bars within each chromosome show the locus of SNPs in the chromosome. **b** Number of MTAs attributed to each chromosome. **c** Annotation results of significant SNPs at −log_10_ *P* > 5. **d** Clustering of important MTAs based on their gene position and *P* value (the label color shows the important gene groups related to flower development).

We used 65 318 522 population SNPs in GWAS to identify 4694 significant marker–trait associations (MTAs) for 14 flower traits at −log_10_ *P* > 5 (Fig. 4a). Therefore, −log_10_ *P* > 5 was selected as a genome-wide threshold for significance based on 4694 SNPs, which was greater than the effective number of independent markers [14]. The highest numbers of MTAs were detected on chromosome 1 (531) and chromosome 2 (451), while the lowest numbers of MTAs were present on chromosome 13 (125) and chromosome 14 (119) (Fig. 4b). Of the 4694 SNPs, 389 MTAs were significant at *P* < 10^−8^, including 88 highly significant MTAs at *P* < 0.0000000001 (10^−10^) (Supplementary Data Table S3). Of the 4694 loci, 3070 were located in the coding sequence (CDS), intron, or 3′/5′-UTR regions of the 1802 genes that were functionally annotated. Among them, 787 functionally annotated genes were involved in 22 KEGG pathways (Supplementary Data Table S4), with the two most enriched pathways related to BRITE hierarchies and protein families: metabolism (Supplementary Data Fig. S2a). The 162 GO terms were divided into three classes, comprising biological processes (138 terms), molecular functions (21 terms), and cellular components (3 terms) (Supplementary Data Table S5; Supplementary Data Fig. S2b). Based on Nr annotation, the highest number of SNP-related genes were annotated as zinc finger proteins (78), followed by pentatricopeptide repeat-containing proteins (63) and receptor-like serine/threonine-protein kinases (48) (Fig. 4c). Cytochrome-P450s and E3 ubiquitin-protein ligases were each associated with 41 genes.

**Table 1:** Statistical variation among 14 phenotypic traits of *C. sinense*.

**Trait**	**Mean**	**Standard deviation**	**Minimum value**	**Maximum value**	**Coefficient of variation**	**Diversity index**
NF	7.90	2.74	2	16	0.35	5.03
NT	6.28	1.5	3	20	0.24	5.07
VDF	3.59	1.27	0.47	7.59	0.35	5.03
TDF	4.53	1.07	0.51	7.06	0.24	5.06
FLWR	0.89	0.42	0.37	5.64	0.48	5.03
LSL	2.72	0.62	0.49	4.09	0.23	5.06
LSW	0.68	0.59	0.28	5.71	0.86	4.92
LSLWR	4.81	1.47	0.53	10.01	0.31	5.04
PL	2.34	0.42	0.57	3.34	0.18	5.08
PW	0.81	0.44	0.21	5.98	0.54	5.02
PLWR	3.11	0.77	0.34	6.51	0.25	5.06
LL	1.34	0.38	0.26	2.65	0.28	5.05
LW	0.94	0.25	0.17	2.26	0.27	5.06
LLWR	1.48	0.58	0.62	5.58	0.39	5.03

Moreover, we found 166 potential MTAs related to flower development and flower organogenesis, which were mainly situated on CDs, intron, or 3’/5’ UTR regions (Fig. 4d), indicating a potential transcript and/or translate-level regulation. Based on functional annotation, we found the most prominent MTAs were associated with MADS-box genes, auxin pathway genes, MYB transcription factors, and ethylene regulators. Among them, we found 37 potential MTAs for NT (Supplementary Data Table S6). The phenotypic variance explained (PVE) for these MTAs ranged from 6.11% on chromosome 18 to 22.12% on chromosome 9. We found 30 significant MTAs for flower size (Supplementary Data Table S6) and 24 of these MTAs showed PVE >10%. For sepal shape, 21 MTAs were found, with 14 MTAs showing PVE >10% (Supplementary Data Table S6). Among the 29 MTAs for lip shape, 21 had PVE >10% (Supplementary Data Table S6). The highest number of significant MTAs (46) was observed for petal shape and the PVE of 36 MTAs ranged from 10.77% on chromosome 6 to 20.84% on chromosome 8 (Supplementary Data Table S6).

### Candidate genes related to individual flower traits

#### GWAS on the trait of tepal number

**Figure 5:**
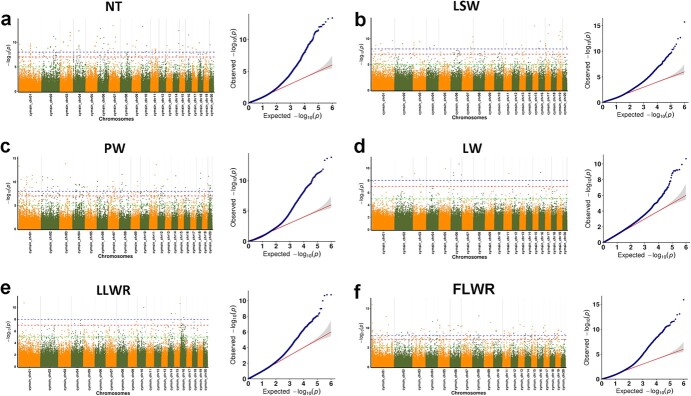
Manhattan plots of −log_10_ *P* versus chromosomal position of MTAs associated with flower traits and quantile–quantile (QQ) plots in *C. sinense*, including number of tepals (**a**), lateral sepal width (**b**), petal width (**c**), lip width (**d**), lip length to width ratio (**e**), and flower length to width ratio (**f**). In Manhattan plots, the green line represents the significant threshold (−log_10_*P* > 5) value, which was determined using a formula based on the marker-based heritability. The red line represents the value corresponding to 0.1/SNP marker number. The blue line is made up of different blue dots, each of which represents the actual observed *P*-value of each SNP, usually expressed as −log_10_ *P*. The straight line indicates the distribution of SNPs under the null hypothesis.

We detected most significant SNPs related to the trait of NT (12 loci with −log_10_*P* > 8) (Fig. 5a). Nine of the top 10 associated SNPs (−log_10_ *P* > 10) were located in the 5′-UTR position of a transcriptional regulator called RABBIT EARS (RBE). Among the other genes related to NT were MADS-box transcription factors (TFs), auxin regulators, and ethylene-responsive TFs (Supplementary Data Table S6).

#### GWAS on the trait of sepal shape

Sepal shape was related to 21 MTAs, including 6 LSW-related MTAs (Fig. 5b), 7 LSL-related MTAs (Supplementary Data Fig. S3a), and 8 LSLWR-related MTAs (Supplementary Data Fig. S3b). Here, the two highly significant MTA genes included Mol001137.gene (MYBS3-like) and Mol024028.gene (MADS-box protein), with −log_10_ *P* values of 8.5 and 8.22, respectively. Both were located in the 3′-UTR region of chromosomes 7 and 4, respectively (Supplementary Data Table S6). In addition, we found seven MADS-box TFs, two MYBs and one auxin regulator related to the trait of sepal shape (Supplementary Data Table S6).

#### GWAS on the trait of petal shape

The highest number of significant MTAs (46) were observed for petal shape (Supplementary Data Table S6), including 41 PW-related MTAs (Fig. 5c), 3 PL-related MTAs (Supplementary Data Fig. S3c), and 2 MTAs associated with PLWR (Supplementary Data Fig. S3d). The four most significant MTAs included Mol009479.gene (DNA-directed RNA polymerase II RPB1), Mol027373.gene (mannose-specific lectin), Mol020177.gene (bHLH), and Mol017032.gene ((S)-coclaurine *N*-methyltransferase) with −log_10_ *P* values of 8.97, 8.86, 9.31, and 3.03, respectively (Supplementary Data Table S6). The first two MTAs were CDSs located on chromosomes 7 and 2, respectively. The third one was intron-based, located on chromosome 20, and the fourth one was 5′-UTR-based, located on chromosome 6 (Supplementary Data Table S6).

#### GWAS on the trait of lip shape

Among the 29 MTAs for lip shape, 17 were related to LW (Fig. 5d), 7 were connected with LLWR (Fig. 5e), and 5 were associated with LL (Supplementary Data Fig. S3e). The three highly significant MTA genes were Mol000239.gene (cyclin-D3-3-like), Mol000238.gene (flowering time control protein, FPA) and Mol025387.gene (AGL61), with –log_10_ *P* values of 9.09, 9.09 and 7.25, respectively (Supplementary Data Table S6). The first two MTAs were located on chromosome 3, while the third one was located on chromosome 4. Annotation analysis showed the abundance of MADS-box TFs and auxin pathway genes.

#### GWAS on the trait of flower size

Flower size was estimated in terms of VDF, TDF, and FLWR, and 30 important MTAs were found to be related to flower size. Most of them were associated with FLWR (24 MTAs, Fig. 5f) compared with VDF (Supplementary Data Fig. S3f) and TDF (Supplementary Data Fig. S3g), each containing 3 MTAs. *ANP1* (Mol021424.gene) and *IAA4-like* (Mol006276.gene) were the most significant MTA genes, with *P* values of 1.7079 × e^−11^ and 3.91 × e^−11^, respectively (Supplementary Data Table S6). Among the other genes, there were six auxin pathway genes, four flowering genes, five MADS-box TFs, three bHLHs and two MYB TFs.

### Significant marker–trait associations obtained from InDels

**Figure 6:**
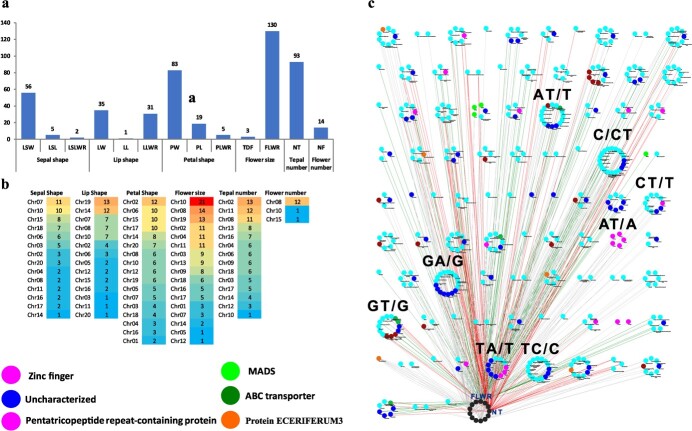
Distribution of InDels among 14 floral traits of *C. sinense* (**a**); chromosome-wise distribution of InDels at −log_10_ *P* > 5 (**b**); and clustering of 477 InDels at −log_10_ *P* > 5 (**c**). The red and green edges show the genes associated with NT and FLWR, respectively. The circular clusters show the alleles and different node colors show the most abundant protein groups associated with InDels.

In addition to the SNPs, we identified 3 906 176 InDels from 675 761 SLAF tags in 195 accessions. Using −log_10_ *P* > 5 as threshold, 477 significant marker-trait-associated InDels were obtained (Supplementary Data Table S7). Of these, sepal shape occupied 63 InDels, including 56 LSW-related, 5 LSL-related, and 2 LSLWR-related InDels (Fig. 6a). Most of these InDels were located on chromosomes 7 (11 InDels), chromosome 10 (10 InDels), chromosome 15 (8 InDels), and chromosome 18 (7 InDels) (Fig. 6b). A total of 67 InDels were associated with lip shape, including 35 for LW, 1 for LL, and 31 for LLWR (Fig. 6a). The major distribution sites were located on chromosome 19 and chromosome 14, containing 13 and 12 InDels, respectively, while each of chromosomes 7, 8, and 10 contained 7 InDels (Fig. 6b). Petal shape was linked with 107 InDels, involving 83 InDels for PW, 19 for PL, and three for PLWR (Fig. 6a). Chromosome 2 contained the most petal shape-related InDels (12), followed by chromosomes 6, 15, and 17, each containing 10 InDels (Fig. 6b). The highest number of InDels (133) was concomitant with flower size, including 130 for FLWR and only 3 for TDF (Fig. 6a). Among these, 21 InDels were located on chromosome 10, while chromosomes 8 and 19 contained 14 and 13 InDels, respectively (Fig. 6b). Among the 93 InDels related to NT, 13 were nested on chromosome 2, 12 occupied chromosome 11, and chromosome 8 contained 11 InDels (Fig. 6b). Only 14 InDels were associated with NF (Fig. 6a); they were placed on three chromosomes, with chromosome 8 containing 12 InDels (Fig. 6b).

**Table 2:** SNP distribution on 20 chromosomes.

**Chromosome**	**No. of SNPs**	**Coverage (Mb)**	**Marker interval (Mb)**	
			**Average**	**Maximum**
cymsin_chr01	6 632 474	376.78	0.000057	0.069
cymsin_chr02	6 504 680	306.08	0.000047	0.037
cymsin_chr03	4 483 176	223.18	0.000050	0.054
cymsin_chr04	4 758 863	205.48	0.000043	0.032
cymsin_chr05	4 382 046	200.82	0.000046	0.039
cymsin_chr06	4 292 808	185.9	0.000043	0.035
cymsin_chr07	3 999 725	189.71	0.000047	0.048
cymsin_chr08	4 191 264	193.45	0.000046	0.069
cymsin_chr09	3 656 891	157.94	0.000043	0.050
cymsin_chr10	3 386 556	151.88	0.000045	0.038
cymsin_chr11	3 283 772	142.31	0.000043	0.035
cymsin_chr12	2 786 779	122.62	0.000044	0.027
cymsin_chr13	2 399 888	110.62	0.000046	0.045
cymsin_chr14	2 604 141	109.96	0.000042	0.034
cymsin_chr15	2 314 964	99.18	0.000043	0.028
cymsin_chr16	2 393 937	108.02	0.000045	0.032
cymsin_chr17	2 508 328	101.52	0.00004	0.049
cymsin_chr18	2 141 477	94.5	0.000044	0.037
cymsin_chr19	2 125 709	93.76	0.000044	0.032
cymsin_chr20	1 540 306	72.95	0.000047	0.033
Total	70 387 784	3246.66	/	/

**Table 3:** InDel distribution on 20 chromosomes

**Chromosomes**	**No. of indels**	**Coverage (Mb)**	**Marker interval (Mb)**	
			**Average**	**Maximum**
cymsin_chr01	289 192	376.78	0.00130	0.1200
cymsin_chr02	324 381	306.08	0.00094	0.0704
cymsin_chr03	211 373	223.18	0.00105	0.0752
cymsin_chr04	249 151	205.48	0.00082	0.0563
cymsin_chr05	233 987	200.82	0.00086	0.0701
cymsin_chr06	228 211	185.90	0.00081	0.0545
cymsin_chr07	252 840	189.71	0.00075	0.0514
cymsin_chr08	251 842	193.45	0.00077	0.0689
cymsin_chr09	150 137	157.94	0.00105	0.0681
cymsin_chr10	163 574	151.88	0.00093	0.0556
cymsin_chr11	199 537	142.31	0.00071	0.0906
cymsin_chr12	156 707	122.62	0.00078	0.0527
cymsin_chr13	135 986	110.62	0.00081	0.0850
cymsin_chr14	140 655	109.96	0.00078	0.1232
cymsin_chr15	124 000	99.18	0.00080	0.0549
cymsin_chr16	139 349	108.02	0.00077	0.0855
cymsin_chr17	177 503	101.52	0.00057	0.0476
cymsin_chr18	117 558	94.50	0.00080	0.0631
cymsin_chr19	113 146	93.76	0.00083	0.0878
cymsin_chr20	85 082	72.95	0.00086	0.0595
Total	3 744 211	3246.68	/	/

Zinc finger proteins were the most abundant among the annotated significant InDels identified at −log_10_ *P* > 5, followed by pentatricopeptide repeat-containing proteins, MADS-box TFs and ABC transporters (Supplementary Data Table S7). However, a number of InDels were uncharacterized. The key allelic sites included GA/G, C/CT, AT/T, GT/G, TA/T, and TC/C, which were shared by most of the InDels (Fig. 6c).

We isolated 31 potential InDels out of 477 (Supplementary Data Table S8). Among these, 7 were related to lip size, 9 were associated with petal size, 4 were related to NT, 10 were FLWR-related InDels, and one InDel was connected with LSW. The most important InDel-associated genes included four GTE9-like TFs identified for LW and PL. Another PL-related InDel (ATGTATG/A) was associated with zinc metalloprotease EGY2 (Supplementary Data Table S8). Associated with PW, another allele (C/AC) was found to be responsible for flowering time control protein FPA and an important allele (TA/A) was associated with zinc finger domain CCCH.

**Figure 7:**
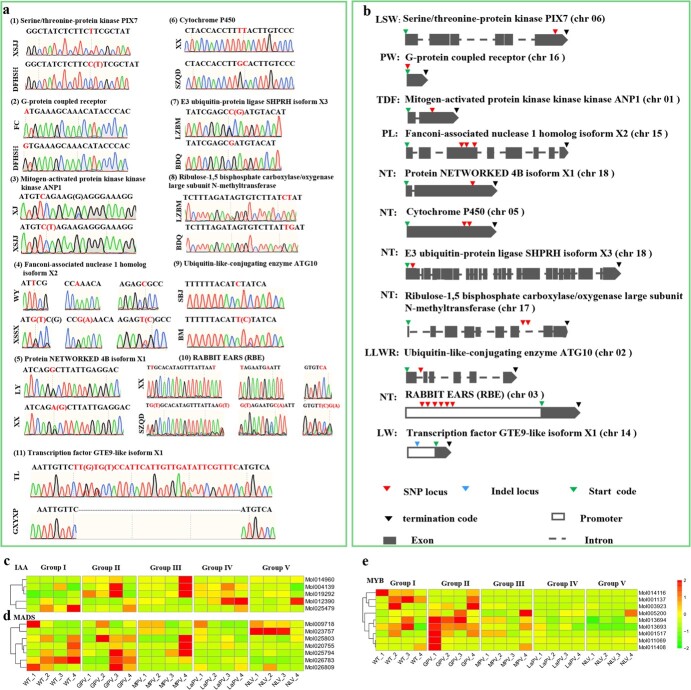
**a** DNA sequence chromatograms of 20 candidate SNPs/InDels. **b** SNP and InDel loci in 10 genes identified by an association analysis. Cultivars used: XSJJ, Xiashanjinju; DFHSH, Dongfanghongshenhe; FC, Fucui; XJ, Xiju; WY, Wangyue; XSSX, Xiashansanxing; LY, Lvyun; XX, Xiaoxiang; SZQD, Shenzhouqidie; LZBM, lvzhuabaimo; BDQ, Baodaoqi; SBJ, Shibajiao; BM, Baimo; TL, Tianlong; GXYXP, Guoxiangyuanxinpin. **c**–**e** Gene expression patterns of auxin-related genes, MADS-box genes, and MYB transcription factors found among the MTAs, respectively [the five varieties included normal flower type (WT), genostemium-like perianth variety (GPV), multi-perianth variety (MPV), labellum-like perianth variety (LaPV), and null-lip variety (NLV)]. Numbers 1 to 4 represent individual floral organs, including sepal, petal, labellum, and genostemium.

**Figure 8:**
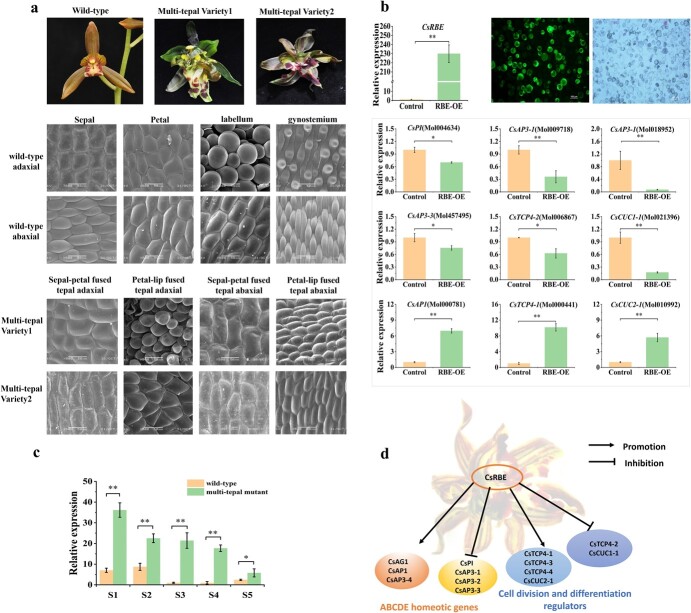
**a** Scanning electron microscope observation of wild-type and mutant flowers. Scale bar = 100 μm. **b** RT–qPCR analysis of *CsRBE* gene at five flower development stages. **c** High expression of *CsRBE* gene in protoplasts from *C. sinense* leaves. Fluorescence observation and RT–qPCR analysis of related genes. **d** Hypothetical regulatory network of *CsRBE* gene in *C. sinense.* Error bars indicate standard deviation. Significance is shown at *P* ≤ .05 (*) and *P* ≤ .01(**).

### Verification of MTAs obtained from SNPs and InDels

We randomly selected 20 MTA loci related to LSW, PW, TDF, PL, NT, LLWR, and LW, and verified the related SNPs and InDels by Sanger sequencing (Fig. 7). For each trait, two germplasms with significant differences were selected from 195 *C. sinense* resources as test materials. The 50-μl PCR products were detected and confirmed by agarose gel electrophoresis, and then directly sequenced. Associated with LSW, serine/threonine-protein kinase PIX7 was tested for XSJJ and DFHSH cultivars. The homozygous allele (T) was evident in XSJJ, while DFHSH was heterozygous for C/T. In the case of PW, an SNP was verified for G-protein coupled receptor, which was homozygous in both FC and DFHSH.

One SNP was verified for TDF. Here, mitogen-activated protein kinase ANP1 expressed a homozygous (C) SNP for XJ and a heterozygous SNP (C/T) for XSJJ. Five SNPs were verified for NT. Protein NETWORKED 4B isoform X1 was both homozygous (G) for LY and heterozygous (A/G) for XX. Two adjacent SNPs (TT/GC) were identified for the cytochrome P450 gene. In the normal cultivar of XX, the allele was homozygous for TT, while in the cultivar with multiple tepals (SZQD) the allele was homozygous for GC. Similarly, two adjacent pairs were verified for ribulose-1,5 bisphosphate carboxylase/oxygenase large subunit *N*-methyltransferase, wherein homozygous alleles CT and TG were observed in LZBM and BDQ, respectively. E3 ubiquitin-protein ligase SHPRH was heterozygous for G/C and homozygous for G. Moreover, GWAS identified several contiguous SNPs associated with NT in the promoter of *RBE* gene. Therefore, *RBE* was also verified for XX and SZQD. Six SNP loci were found to be different in two varieties, and in the cultivar with multiple tepals (SZQD), six SNP loci were heterozygous. One deletion was also confirmed in the TF GTE9-like situated on LW. Figure 7b shows the location of these loci, which indicated that the MTAs obtained from GWAS were reliable.

Most loci were located in the 3′-UTR and 5′-UTR positions, indicating a potential transcript and/or translation-level regulation. We thus determined the correlation between gene expressions of these loci and phenotypic variations. We sequenced the transcriptome of 20 flower organs from five representative varieties with significant differences in flower organ size and morphological structure, namely normal flower type (WT), genostemium-like perianth variety (GPV), multi-perianth variety (MPV), labellum-like perianth variety (LaPV), and null-lip variety (NLV). We identified 27 locus-linked genes, including MADS-box genes, auxin pathway genes, and MYB genes, which were further verified. As shown in Fig. 7c, three out of five AUX/IAA genes were highly expressed in the fused genostemium of multi-tepal flowers with continuous floral organ division. MADS-box genes were divided into two groups. A pair of MADS-box genes in group I showed contrasting expression patterns in the sepals of WT and NLV, suggesting that this pair might be related to sepal development (Fig. 7d). The genes in group II were differentially expressed in individual floral organs of normal flowers, and the differential expression disappeared in the varieties with floral organ fusion or transformation, indicating their important role in flower development. Among the nine MYBs, the expression level of MYBs was low in NLV and LaPV, while only one gene showed high expression in the genostemium of MPV (Fig. 7e). However, in GPV where the genostemium expanded to the perianth, all the MYBs showed high expression, showing significant organ-specific expression, with four concentrated in the sepals and two in the genostemium.

### RABBIT EARS is an important regulator of the multi-tepal trait in *C. sinense*

The wild flowers showed variations in the epidermal cells of genostemiums, lips, petals and sepals, presenting a polygonal to papillae/cupola/conical shape. In contrast, multi-tepal varieties developed sepal–petal or petal–lip fused structures. Scanning electron microscopy of adaxial and abaxial surfaces of floral organs suggested significant variations between wild-types (Fig. 8a, upper two rows) and multi-tepal mutants (Fig. 8a, lower two rows). Although it is well known that multi-tepal mutations are directly related to the expression of C-class MADS-box *AGAMOUS* (*AG*) genes, our GWAS analysis did not find any loci linked to *AG* orthologous genes. Interestingly, out of the 12 loci associated with the multi-tepal trait with the highest *P*-value, 8 were associated with the 5′-UTR of the *RBE* gene, ranging from 4630 to 4973 upstream of ATG. Gene expression levels were analyzed in wild-types and different multi-tepal variants. The results indicated that the *RBE* gene was concentrated in the early floral developmental stages (Fig. 8b). The gene expression level was significantly increased in multi-tepal flowers compared with wild-type through all developmental stages (Fig. 8b), indicating that this gene might be closely related to multi-tepal variation.

We cloned the *CsRBE* gene of *C. sinense*, with a length of 678 bp. Phylogenetic analysis revealed that only 62% of *CsRBE* sequences were consistent with *A. thaliana* (Supplementary Data Fig. S4). Considering that the *CsRBE* gene has not been reported in other species except *A. thaliana*, we further analyzed the potential role of *CsRBE* in the regulation of flower-related genes via efficient transient overexpression in *C. sinense* protoplasts. The *CsRBE* gene was 230 times more highly expressed in protoplasts (Fig. 8b), and the expression of 18 floral-related genes was detected to be up- or downregulated. Among them, the expression of *CsPI*, *CsAP3-1*, *CsAP3-2*, *CsAP3-3*, *CsTCP4-2*, and *CsCUC1-1* was decreased by 24.5–93.1%. The downregulation of *CsAP3-2* was the most significant, followed by *CsCUC1-1*, while the expression of *CsAP1*, *CsAG1*, *CsAP3-4*, *CsTCP4-1*, *CsTCP4-3*, *CsTCP4-4*, and *CsCUC2-1* was increased by 1.3–10.17 times, and the increase of *CsTCP4-1* expression was the most significant (Fig. 8b and Supplementary Data Fig. S5).

## Discussion

Flower size and tepal morphology are considered the most classical esthetic traits of ornamental plants [15]. Flower structure and organ shape are the major determinants of reproductive behavior and the domestication process of plants [16]. *Cymbidium sinense*, being one of the dazzling and remunerative orchids, has a widespread geographical diversity in China. Natural selection and non-natural domestication have engineered a wide germplasm diversity, mainly in flower number and floral organ shape. We sequenced 195 *C. sinense* accessions with diverse morphological variations of flower count, sepal size and shape, lip size and shape, and petal size and shape (Fig. 1a). From these accessions, we estimated the correlations among 14 traits related to flower size and organ morphology and found significant positive correlations among most of the groups (Fig. 1b). This outcome makes sense in that flower size and shape are potentially affected by the length and width of each flower organ. For example, the disk area in sunflower is positively correlated with disk diameter [17]. Moreover, significantly positive correlations have been observed in sepal length and sepal width, sepal width, and style width, as well as sepal width and petal width [18]. A total of 12 traits related to flower size (width, length and area of flower, lip, sepal, and petal) were observed in 117 *F*_1_ progenies of *Phalaenopsis intermedia* (cross between *P. equestris* and *P. aphrodite*) [12]. In our study, most of the traits were positively correlated. However, some significantly negative correlations existed among LSW, PW, and LW, suggesting that organ width may vary independently among floral organs. The traits also showed a potential range of variations in their levels (Table 1). A relatively high coefficient of variation was associated with LSW, PW, FLWR, and LLWR. Especially, the variation coefficient was as high as 0.86 for LSW, which may indicate that the artificial selection of *C. sinense* is based on different LSW levels. Floral traits, such as shape, color, fragrance, and size are potentially under selection due to pollinator predilections [19]. PCA and population genetic structure and phylogenetic analyses divided the 195 accessions into two distinct clusters (Fig. 3). Analysis of genome-wide LD decay of all the collections justified a low LD level in the genome at a significant *R*^2^ < .1 (Fig. 3c), which is statistically reliable.

Since the application of GWAS to investigate the human retina [20], it has been used to analyze important traits in different plants, such as *A. thaliana*, *Sorghum bicolor*, *Triticum aestivum*, *B. napus*, *Z. mays*, *Hordeum vulgare*, *Gossypium hirsutum* and *Camellia sinensis* [21–23]. Genetic maps have been constructed for some important orchids, such as *Phalaenopsis*, *Dendrobium*, and *Vanilla* [15]. A cross between *Dendrobium officinale* and *D. aduncum* generated 349 polymorphic loci [24]. In *P. aphrodite*, a genetic map was constructed using 2905 SNP markers [25], while 1191 SNPs have been identified in *P. equestris* from 117 progenies [15]. However, no analysis of orchid trait associations in natural populations has been conducted yet, and no GWAS or SNPs have been documented on *Cymbidiums*, which are elite flowers representing the most versatile orchids in the world. We noted 4694 significant MTAs for 14 floral traits of *C. sinense* at −log_10_ *P* > 5 (Fig. 4a).

GWAS is an outstanding technique to recognize genomic loci allied with preferred traits [26]. Recognizing the location and number of trait-related loci is imperative to plan successful breeding strategies. Of the 4694 SNP MTAs, 3070 loci were located in the CDS, intron, or 3′/5′-UTR regions of the genes. Annotation of these SNP-related genes suggested a number of genes related to flowering regulation, hormonal pathways including auxin, cytokinin, gibberellin, and ABA, and cell division (Supplementary Data Table S5). For example, out of 29 MTAs for lip shape, Mol000239.gene (cyclin-D3-3-like), Mol000238.gene (flowering time control protein FPA), and Mol025387.gene (AGL61), with −log_10_ *P* values of 9.09, 9.09, and 7.25, respectively, were the most important SNPs. Thirty flower size-associated MTAs contained ANP1 (Mol021424.gene) and IAA4-like (Mol006276.gene) as the most significant SNPs, with *P* values of 1.7079 × e^−11^ and 3.91 × e^−11^, respectively.

A total of 477 potential InDels–MTAs were identified at log_10_ *P* > 5 (Supplementary Data Table S7), including 63 for sepal shape, 67 for lip shape, 107 for petal shape, 133 for flower size, 93 for tepal number, and 14 for flower number (Fig. 6a). Among these, zinc finger proteins were the most abundant, followed by pentatricopeptide repeat-containing proteins, MADS-box TFs, and ABC transporters (Fig. 6c). The most important InDel-associated genes included four GTE9-like TFs identified for LW and PL. GTE9 is a Global Transcription Factor Group E protein that interacts with BT2 (BTB-domain protein) to mediate sugar and ABA responses in *A. thaliana* [27]. BT2 controls responses to various stresses, hormones, and metabolic pathways in *A. thaliana*. Another PL-related InDel (ATGTATG/A) was associated with zinc metalloprotease EGY2 (Supplementary Data Table S7). It is an ethylene-dependent gravitropism-deficient and yellow-green2 metalloprotease, which plays a role in hypocotyl elongation and regulates the expression levels of nuclear and plastid-encoded genes [28]. Associated with petal width, an allele (C/AC) was found to be associated with flowering time control protein FPA, which works through the circadian clock pathway and regulates flowering time in a number of plants [29]. Moreover, a vital allele (TA/A) was associated with zinc finger domain CCCH, which plays various roles in plant growth and stress responses [30].

Although candidate SNP and InDel loci have been mined through GWAS, some false-positive alleles may be erroneously associated with QTLs due to LD attenuation, different sample sizes, population structures, the number of molecular markers, and analytic methods [31]. Therefore, key SNPs should be verified through functional verification or by associating them in multiple groups [23]. We selected 20 SNPs/InDels for verification (Fig. 7). Serine/threonine kinases form a crucial network in plant cells, acting as a CPU (central processing unit) that perceives information from receptors that detect phytohormones and environmental conditions, and converts them into useful changes in gene expression, cell cycle, metabolism, and cell growth [32]. Serine/threonine kinases were among the highly annotated SNP-related genes (Fig. 4c). We confirmed the SNP position at the exon of chromosome 6 (Fig. 7b). Similarly, GPCRs (G-protein coupled receptors) represent the transmembrane receptors transducing external environmental signals inside the cell [33]. They involve plant defense responses, stomatal regulation, seed germination and growth and the genesis of plant organs, such as rosette leaf, root, silique, and flower [34, 35]. Mitogen-activated protein kinases function downstream of receptors/sensors and regulate normal plant growth and its adaptation to fluctuations of the environment by coordinating cellular responses [36]. Cytochrome P450 enzymes have extensive roles in plant growth, petal development, flower organogenesis, and floral JA and GA homeostasis [37, 38]. Most of the verified SNPs were located on the exon positions, except for ribulose-1,5 bisphosphate carboxylase/oxygenase large subunit *N*-methyltransferase, present on the intron of chromosome 17 (Fig. 7b). This suggests the high efficiency of SNPs in the process of selection.

In the analysis of 14 flower morphological traits, we found that the highest number of variation sites was associated with NT. In particular, we verified six SNP loci in the 5′-UTR region of the *CsRBE* gene, which were significantly associated with the number of tepals, and the phenotypic interpretation rate was 7.853–9.041%. Previous reports in *A. thaliana* indicated that limiting the expression of *RBE* to the petal precursor cells was essential for flower development [39]. *RBE* plays an indispensable role in maintaining the organ primordial boundaries within a whorl as well as homeotic gene expression boundaries between whorls [40, 41]. It regulates the *Arabidopsis* organ boundary regulators, including *CUC1* (*CUP SHAPED COTYLEDON 1*) and *CUC2* by miR164C, and stimulates the growth of petal primordia by a direct and negative regulation of *TCP5* and *TPC4* [42]. The floral homeotic genes, such as *PISTILLATA* (*PI*), *APETALA3* (*AP3*), and *AGAMOUS* (*AG*), are also regulated by *RBE* [39, 40]*.* Analysis of transcriptome data in wild-type and multi-tepal variants also suggested a close relationship between differential gene expression and the multi-tepal phenotype. At the same time, overexpressing the *CsRBE* gene in the protoplast of *C. sinense* caused expression changes of 18 floral-related genes, including *CsAP3*, *CsPI*, *CsTCP*, and *CsCUC* (Fig. 8c, Supplementary Data Fig. S5), indicating its important regulatory role in floral organ development of *C. sinense*. (Fig. 8d). Interestingly, we found that *CsRBE* was acting both as stimulator and inhibitor of the genes regulating cell activities and flower organ development, probably owing to the feedback loop among homologous genes within the gene family. However, the expression regulation mechanism of *CsRBE* and what elements regulate its specific location have not been reported yet. Our results show that the variation of multi-tepal flowers is closely related to *CsRBE*, and the variation at the loci located in the promoter region may be the direct driving force behind the change in its expression pattern. Further study will be required to conduct a detailed analysis of the expression regulation mechanism.

## Materials and methods

### Plant materials and genotyping

The 195 *C sinense* accessions were obtained from various resources (Supplementary Data Table S1). These accessions represent diverse morphological variations of flower count, sepal size and shape, lip size and shape, and petal size and shape. A total of 14 traits were selected for GWAS testing, comprising number of flowers (NF), number of tepals (NT), vertical diameter of flower (VDF), transverse diameter of flower (TDF), flower length to width ratio (FLWR), lateral sepal length (LSL), lateral sepal width (LSW), lateral sepal length to width ratio (LSLWR), petal length (PL), petal width (PW), petal length to width ratio (PLWR), lip length (LL), lip width (LW), and lip length to width ratio (LLWR). DNA was isolated from each accession using flower samples following a modified CTAB (cetyl trimethyl ammonium bromide) method [43]. The DNA pellets were suspended using a T1/10E buffer containing 0.1 mM EDTA, and 10 mM Tris–HCL at a pH of 8.0. DNA quality and quantity were ascertained on a NanoDrop™ One spectrometer (Thermo Fisher Scientific, USA). Finally, the DNA concentration of 50 ng/mL was adjusted for NSP array-based genotyping.

The 195 *C. sinense* accessions were genotyped with the 51 K Axiom^®^*Cymbidium* array. For this, 200 ng per sample of genomic DNA was used. Amplification and random fragmentation of this DNA into lengths of 25–125 bp were performed using the Axiom^®^ 2.0 reagent kit (Thermo Fisher Scientific, USA). Hybridization of DNA fragments to the array was performed with the Affymetrix^®^ GeneTitan system following the manufacturer’s instructions. Hybridization signal processing was done using the CEL files in the Affymetrix^®^ Power Tools software package (v1.18) for SNP calling. Significant SNPs were isolated using the following criteria: missing data <10% and minor allele frequency >5%. Missing data were imputed for the resulting SNPs using BEAGLE (v5) with default parameter settings [44].

### Population structure and association analysis

The *C. sinense* population structures were suggested with the STRUCTURE (v.2.3.4) program [45]. The STRUCTURE model provides the ability to incorporate admixture and interconnected allele frequencies. To determine the best number of clusters (*K*), 10 independent simulations were performed for each 10 *K*s (1–10) with 10 000 iterations as burn-in period and a Markov chain Monte Carlo (MCMC) run length of 10 000 iterations. After the first round, six *K*s (4–9) were used for further simulations with 20 000 iterations as burn-in period and 100 000 iterations as an MCMC run length. The resulting log-likelihood estimates for the *K*s were tested to ascertain the best *K* using the delta *K* method [46]. The best *K*-based membership coefficients of 195 *C. sinense* accessions were used to construct a Q matrix (population structure matrix). Additionally, the R package was used to perform hierarchical clusters. The poppr package (v2.9.4) was used to assess Nei’s genetic distances [47] between *C. sinense* accessions [48], followed by hierarchical clustering analysis using an unweighted pair group method with arithmetic mean (UPGMA).

For the identification of MTAs for 14 flower traits, association analysis was performed using the MLMM (multilocus mixed model) [49] in association with the GAPIT (genomic association and prediction integrated tool) [50]. The kinship matrices and covariates Q were used to lessen the false-positive associations caused by familial relatedness and population structure [51]. The VanRaden algorithm was used to generate the kinship matrix [52]. Significant MTAs were sorted at *P* < 0.0005 and then a genome-wide threshold at *P* < 0.00005 was used, which was ascertained on the basis of effective number of independent markers (*M*_e_) [14]. For *M*_e_ estimation, GEC (Genetic Type I Error Calculator) software (http://pmglab.top/gec/#/) was used for the equation 0.05/*M*_e_. The phenotypic variance explained (PVE) by a significant marker was calculated using the following equation in base R citation:

PVE% = (SS_sig:marker_/(SS_all sig:marker_ + e)) × 100

where SS denotes the sum of squares and e represents the threshold from the ANOVA fitted with a linear model incorporating the phenotypic data and all significant markers [53]. The candidate MTAs were further evaluated using the reference genome of *C. sinense* and the annotation results were obtained.

### Candidate gene identification and enrichment analysis

The significantly associated SNP/InDel loci were functionally annotated. The candidate genes associated with SNP/InDels were annotated using the *C. sinense* reference genome annotation [2]. The GO (Gene Ontology; http://geneontology.org) and KEGG pathway (http://www.genome.jp/kegg) databases were used to assign metabolic attributes to candidate genes.

### Candidate SNP/InDel marker verification

Twenty candidate SNPs/InDels associated with 11 genes were verified. Total DNA was extracted and PCR amplification primers (Supplementary Data Table S9) were designed consistent with the sequences upstream and downstream of the 20 SNP/InDel loci. The amplification products were processed using the SAP digestion system. After the SNaPshot reaction, sequencing was performed and the genotypes of the 20 SNP/InDels were counted.

### Transient protoplast expression assay of *CsRBE*

The transient expression of *CsRBE* was checked in the protoplast of *C. sinense* following our previously established protocol [54]. Briefly, the *CsRBE* CDS was inserted into PAN580-GFP vector and the recombinant vector was introduced into *Escherichia coli* DH5α-competent cells. The bacteria were replicated and the plasmid was extracted and mixed with PEG solution for protoplast transfection. Transient expression was calculated using GFP reporter expression of Pan580-GFP vector. The LSM710 confocal laser scanning microscope was used to measure GFP fluorescence.

### qRT–PCR expression of transient CsRBE and other genes

Protoplasts with the transient CsRBE gene were harvested to measure the expressions of *CsPI*, *CsAP1*, *CsAP3-1*, *CsAP3-2*, *CsAP3-3*, *CsAP3-4*, *CsTCP4-2*, *CsCUC1-1*, *CsCUC2-1*, *CsTCP4-1*, *CsTCP4-3*, *CsTCP4-4*, and *CsAG1* (primers are shown in Supplementary Data Table S10). Using *CsUBQ* as internal standard, qRT–PCR was performed in a Bio-Rad CFX-96 Real-Time PCR System (Bio-Rad, USA) and gene expression was measured by the 2^−ΔΔΤ^ method. Similarly, the qRT–PCR expression of *CsRBE* was measured for five stages of flower development.

### Scanning electron micrography

The surface cell morphology of floral organs was observed through a scanning electron microscope. The dissected pieces were fixed using 2% formaldehyde and 3% glutaraldehyde solution for 24 h, followed by dehydration in acetone, critical-point drying in liquid CO_2_, and mounting on gold-coated stubs and sputter. The samples were observed with a JSM-6360LV (JEOL) scanning electron microscope.

### Statistical analysis

ANOVA (one-way) was used through SPSS software (SPSS, Chicago, USA; v16.0) to measure statistical significance.

## Supplementary Material

Supplementary_figures_R1_uhad206

Supplementary_tables_R1_uhad206

## Data Availability

The original contributions presented in the study are publicly available. The supplementary data are provided along with the manuscript as supplementary tables and supplementary figures. The sequencing clean data have been uploaded to the database of the BIG Data Center (http://gsa.big.ac.cn/index.jsp) under accession number PRJCA020233.
