# Supplementary material for: Genome-wide association analysis identified molecular markers and candidate genes for flower traits in Chinese orchid (Cymbidium sinense)
Source: Hortic Res. 2023 Oct 13;10(11):uhad206. doi: 10.1093/hr/uhad206 (PMC10689080; doi:10.1093/hr/uhad206)
Supplement: Supplementary_figures_R1_uhad206 [file supplementary_figures_r1_uhad206.docx]

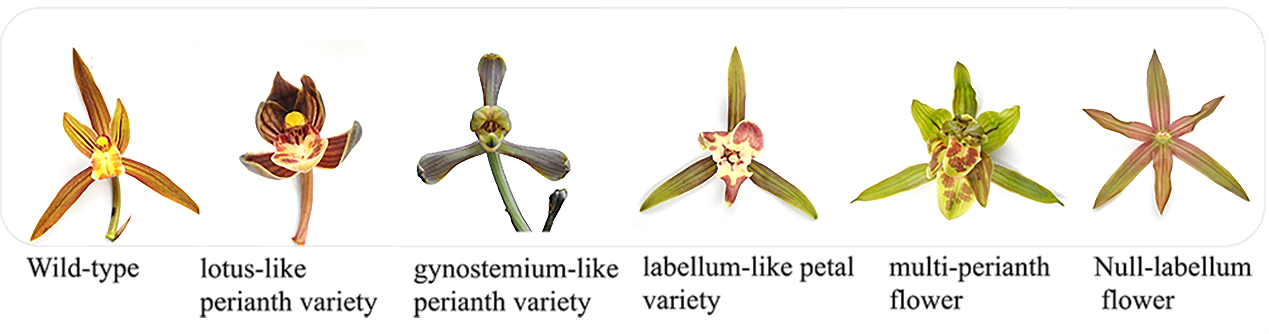


**Figure S1.** Floral organ morphology of *C. sinense*


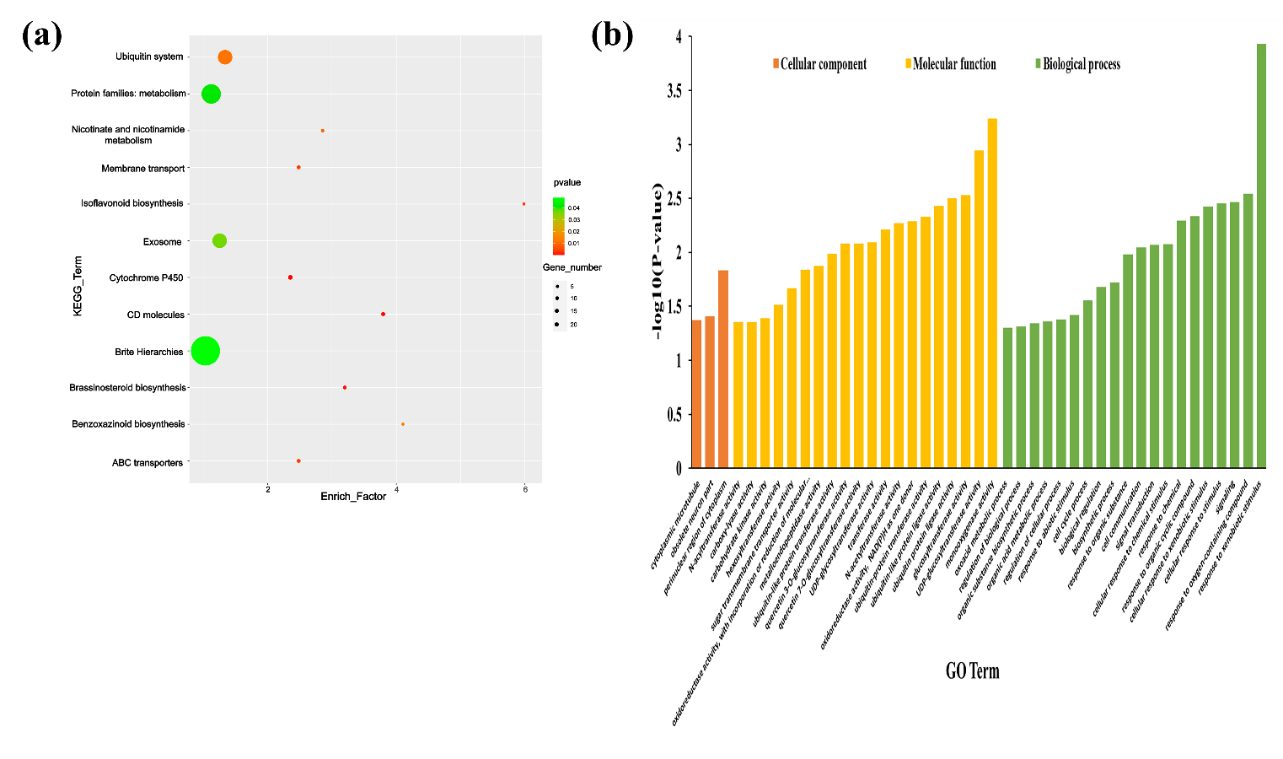


**Figure S2.** KEGG (a) and GO (b) analyses


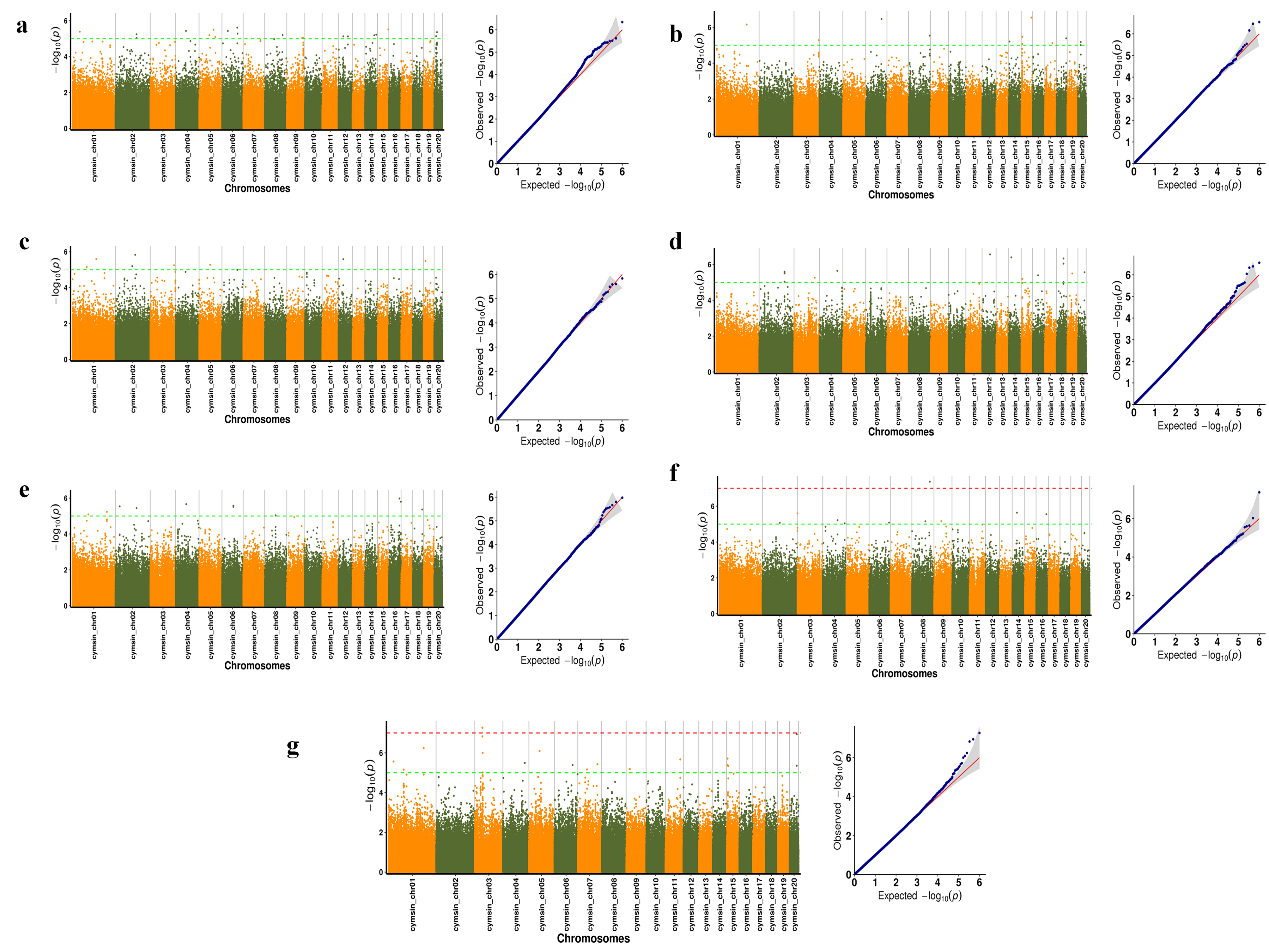


**Figure S3.** Manhattan plots of -Log10 (P) vs. chromosomal position of MTAs associated with flower traits and quantile-quantile (QQ) plots in *C. sinense*, including lateral sepal length (a), lateral sepal length to width ratio (b), petal length (c), petal length to width ratio (d), lip length (e), vertical diameter of flower (f), and transverse diameter of flower (g).


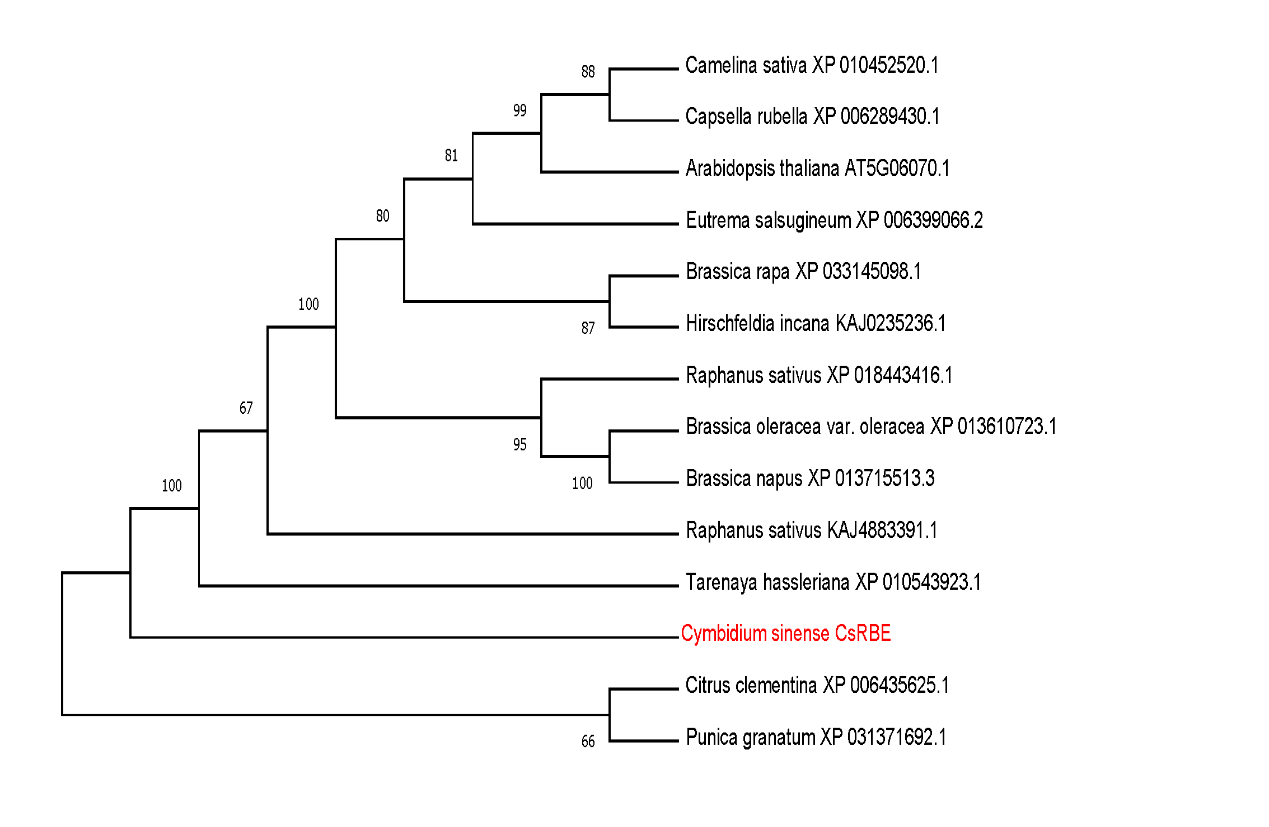


**Figure S4.** Phylogenetic analysis of *RBE* gene.


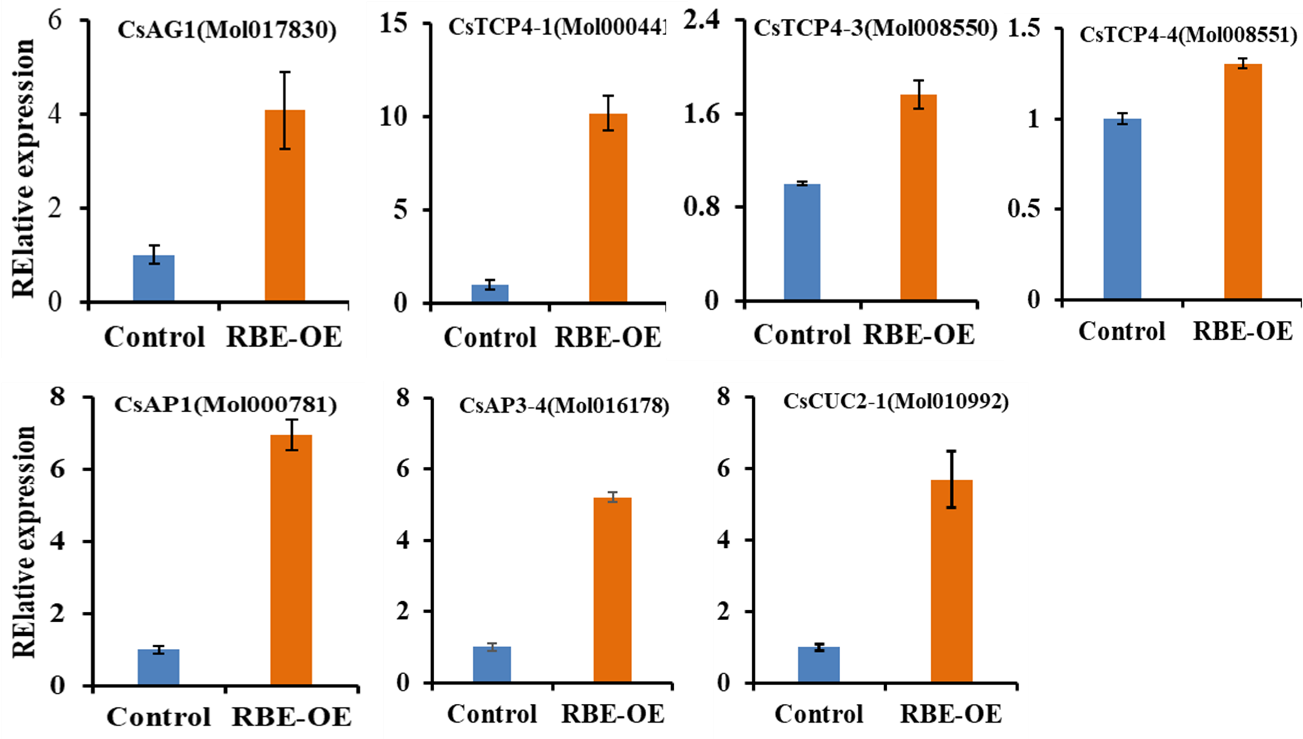


**Figure S5.** High RBE expression in protoplasts from *C. sinense* leaves and RT-qPCR analysis of related genes
